# Structure and Dynamics of Human Chemokine CCL16—Implications for Biological Activity

**DOI:** 10.3390/biom12111588

**Published:** 2022-10-28

**Authors:** Oliver H. Weiergräber, Dušan Petrović, Andreas Kislat, Martin Pattky, Judith Fabig, Renu Batra-Safferling, Jan Schulte am Esch, Karen Hänel, Carolin Huhn, Birgit Strodel, Bernhard Homey, Dieter Willbold

**Affiliations:** 1 Institute of Biological Information Processing, IBI-7: Structural Biochemistry, Forschungszentrum Jülich, 52425 Jülich, Germany; 2 Klinik für Dermatologie, Universitätsklinikum Düsseldorf, 40225 Düsseldorf, Germany; 3 Zentralinstitut für Engineering, Elektronik und Analytik, ZEA-3: Analytik, Forschungszentrum Jülich, 52425 Jülich, Germany; 4 Klinik für Allgemein-, Viszeral- und Kinderchirurgie, Universitätsklinikum Düsseldorf, 40225 Düsseldorf, Germany; 5 Institute of Theoretical and Computational Chemistry, Heinrich-Heine-Universität Düsseldorf, 40225 Düsseldorf, Germany; 6 Institut für Physikalische Biologie und BMFZ, Heinrich-Heine-Universität Düsseldorf, 40225 Düsseldorf, Germany

**Keywords:** CCL16, chemokine, glycosaminoglycan, haptotaxis, limited proteolysis

## Abstract

Human C-C motif ligand 16 (CCL16) is a chemokine that is distinguished by a large cleavable C-terminal extension of unknown significance. Conflicting data have been reported concerning its tissue distribution and modulation of expression, rendering the biological function of CCL16 enigmatic. Here, we report an integrated approach to the characterisation of this chemokine, including a re-assessment of its expression characteristics as well as a biophysical investigation with respect to its structure and dynamics. Our data indicate that CCL16 is chiefly synthesised by hepatocytes, without an appreciable response to mediators of inflammation, and circulates in the blood as a full-length protein. While the crystal structure of CCL16 confirms the presence of a canonical chemokine domain, molecular dynamics simulations support the view that the C-terminal extension impairs the accessibility of the glycosaminoglycan binding sites and may thus serve as an intrinsic modulator of biological activity.

## 1. Introduction

Chemotactic cytokines (chemokines for short) represent a family of secretory proteins typically containing 70–75 residues with two disulphide bonds in their mature forms, which signal via G-protein coupled heptahelical receptors [1]. They play important roles in the activation and directional migration of leukocytes and several other cell types during inflammation, immune surveillance, and development. Based on the arrangement of disulphide bonds, the chemokine family has been divided into four groups, commonly named CC, CXC, CX_3_C, and XC. In the CC-subfamily (alternatively known as the β class), the first two cysteine residues, which engage in the two conserved disulphide bonds, are immediately adjacent; in the CXC (α) and CX_3_C (δ) chemokines, they are separated by one and three residues, respectively. The XC (γ) subfamily represents an exception in that the first cysteine, and thus the first disulphide bond, is missing. As evidenced by biochemical data as well as crystallographic structures, chemokines usually engage their receptors as monomers in a 1:1 stoichiometry [1,2]. It is important to note, however, that most of these molecules form dimers or higher-order oligomers in solution, with preferred arrangements depending on the subtype. Indeed, oligomerisation has been demonstrated to be essential for the induction of migration by certain chemokines in vivo [3]. Another property shared by most, if not all, chemokines is their affinity for glycosaminoglycan (GAG) chains, which are ubiquitous constituents of proteoglycans in the extracellular matrix and on cell surfaces. Each type of GAG is composed of a linear repetitive sequence of characteristic disaccharide units carrying a highly complex sulfation pattern. Association with these acidic polysaccharides will limit the diffusion of chemokine molecules and thus enable the formation of stable haptotactic gradients guiding cell migration [4]. Of note, chemokines often display a strong mutual dependence of GAG binding affinities and oligomericity [5]. The biological activity of these mediators can be further altered by post-translational modifications, with the mechanism most extensively characterised being limited proteolysis [6]. Cleavage of a few residues at the N-terminus of CXC chemokines, for instance, typically enhances receptor activation, whereas in CC-type molecules, a similar modification often gives rise to receptor antagonists.

The C-C motif ligand 16 (CCL16) has been discovered independently by three groups via expressed sequence tag database searches [7,8,9], yielding very different expression profiles. While Shoudai et al. detected CCL16 transcripts exclusively in liver mRNA [7], Hedrick et al. reported widespread expression, with particularly high levels in activated monocytes, but only a weak signal from the liver [8]. In contrast, Youn et al. were unable to detect significant levels of CCL16 expression in any of the tissues tested [9]. The reasons for these discrepancies are unknown at present. As expected for a CC-type chemokine, CCL16 attracts monocytes and lymphocytes in vitro, albeit only at comparatively high concentrations [8]. Based on its amino acid (aa) sequence, the primary CCL16 translation product has been predicted to contain a 23-aa signal sequence followed by a canonical CC-type chemokine domain and an approximately 25-aa C-terminal extension with weak secondary structure propensity. The significance of the latter segment is unclear. Recent evidence indicates that it can be removed by matrix metalloproteinases in vitro, leading to an enhanced glycosaminoglycan binding of the truncated molecule; a more limited processing of the N-terminus has also been observed [10]. Despite these advances, the physiological role of CCL16 has remained obscure. Based on relatively high plasma levels and the lack of obvious regulation thereof, initial reports have questioned the in vivo relevance of leukocyte chemotactic activity for CCL16, favouring a housekeeping role instead. On the other hand, recent evidence has associated this chemokine with a number of inflammatory conditions, such as ulcerative colitis [11], irritable bowel syndrome [12], eosinophilic pneumonia [13], preeclampsia [14], cardiovascular disease [15], and chronic kidney disorders [16]. These observations might be related to the expression of the chemokine in monocytes, which is induced by interleukin (IL)-10 [8].

In an effort to resolve existing ambiguities and gain insight into the function of CCL16 in vivo, we have taken a translational approach by combining biological and biophysical methodologies. Specifically, we have re-examined CCL16 expression in human tissues using state-of-the-art techniques, confirming constitutive synthesis in the liver. The crystal structure of the protein, complemented by the molecular dynamics (MD) simulation of flexible segments, provides important clues as to the regulation of GAG binding, thus offering an explanation for the high plasma levels found in healthy subjects. Based on these findings, a model illustrating the biological life cycle of CCL16 is discussed.

## 2. Materials and Methods

### 2.1. Materials

Normal human liver RNA samples (*n* = 4) were commercially available (Clontech; AMS Biotechnology, Abingdon, United Kingdom); diseased liver tissues with liver cirrhosis, hepatitis B virus (HBV) infection with and without tumour, hepatitis C virus (HCV) infection with and without tumour, cholangiocarcinoma (CCC), and hepatocellular carcinoma (HCC) were obtained from the Universitätstumorzentrum (Heinrich-Heine-Universität Düsseldorf, Düsseldorf, Germany). Recombinant human IL-1β, human IL-6, and human tumour necrosis factor (TNF)-α were obtained from Roche (Mannheim, Germany) and oncostatin M (OSM) from R&D Systems (Wiesbaden, Germany). The BIGE database has been described previously [17,18].

### 2.2. Cell Culture

Human primary epidermal keratinocytes, dermal fibroblasts, and dermal microvascular endothelial cells were cultured in specialised media KGM-2, FGM-2, and EGM-2, respectively (Lonza, Cologne, Germany), as described previously [19,20]. Human primary hepatocytes were isolated and cultured as described [21].

### 2.3. RNA Isolation and Real-Time PCR

RNA was extracted from primary cells and normal human liver using the RNeasy Mini Kit (Qiagen, Hilden, Germany) according to the manufacturer’s protocol. RNA was reverse-transcribed using standard protocols. Gene expression was detected by TaqMan Universal PCR Master Mix with a CCL16 probe Hs00171123_m1 (Thermo Fisher Scientific, Bremen, Germany) on an ABI Prism 7000 Sequence Detection System (Applied Biosystems, Weiterstadt, Germany). CCL16 expression was normalised to the expression of 18S rRNA (TaqMan Ribosomal RNA Control Reagents, Thermo Fisher Scientific, Bremen, Germany).

### 2.4. Immunohistochemistry

Frozen liver sections were fixed in acetone and immunostained as described [19]. Sections were stained with mouse anti-human CCL16 monoclonal IgG1 antibody (R&D Systems, Wiesbaden, Germany), using appropriate isotype antibodies as negative controls. Sections were counterstained with hematoxylin.

### 2.5. Cytokine Analysis

CCL16 expression was quantified by a protein ELISA according to the manufacturer’s protocol (R&D Systems, Wiesbaden, Germany) in sera from patients before partial liver resection and at various time points afterwards.

### 2.6. Heparin Affinity Chromatography

Heparin binding proteins were enriched using HiTrap Heparin HP (GE Healthcare Life Sciences, Solingen, Germany), according to the manufacturer’s instructions. Briefly, the serum was isolated using BD Vacutainer SST II Advance tubes (Becton Dickinson, Heidelberg, Germany), filtered, and diluted 1:1 in binding buffer (50 mM Tris-HCl, 50 mM sodium chloride, pH 7.4). The columns were washed, the serum was applied, and the columns were washed again. Bound proteins were eluted with 50 mM Tris-HCl, 2 M sodium chloride, pH 7.4.

### 2.7. Immunoblotting

Enriched heparin binding proteins were centrifuged to pellet debris, separated by NuPAGE Novex 4–12% [bis(2-hydroxyethyl)amino]tris(hydroxymethyl)methane gels (Thermo Fisher Scientific, Bremen, Germany), transferred to a nitrocellulose membrane (Amersham Protran Premium, GE Healthcare Life Sciences, Solingen, Germany), and analysed by immunoblotting. CCL16 was detected by a biotinylated polyclonal goat anti-human CCL16 antibody and streptavidin-coupled horseradish peroxidase (both R&D Systems, Wiesbaden, Germany). Amersham ECL Prime Western Blotting Detection Reagent (GE Healthcare Life Sciences, Solingen, Germany) was used as a substrate.

### 2.8. Statistical Analysis

Unpaired Mann–Whitney U tests were performed using GraphPad Prism v. 5.03 (GraphPad Software, San Diego, CA, USA). *p* values < 0.05 were considered statistically significant.

### 2.9. Protein Expression and Purification

A codon-optimised synthetic cDNA coding for mature human CCL16 was obtained from Life Technologies (Darmstadt, Germany) and ligated into a modified pET-15b vector using PspOMI and XhoI restriction sites. *E. coli* SHuffle T7 cells (New England Biolabs, Frankfurt, Germany) were transfected with the resulting plasmid and grown in LB media containing 1 mM ampicillin at 30 °C until the OD_600_ reached 0.3. Following induction with 1 mM isopropyl β-D-thiogalactopyranoside, protein expression was allowed to proceed for 4–6 h. Cells were sedimented by centrifugation (20 min at 5000× *g*), resuspended in cold lysis buffer (50 mM sodium dihydrogen phosphate, 500 mM sodium chloride, 10% (*v/v*) glycerol, and EDTA-free protease inhibitor cocktail (Roche, Mannheim, Germany), pH 8.0 via sodium hydroxide), and disrupted by sonication. Lysates were cleared by centrifugation (20 min at 50,000× *g*) and subjected to metal affinity chromatography using Ni-NTA agarose (Qiagen, Hilden, Germany) in batch mode. After incubation, beads were washed with lysis buffer additionally containing 5–20 mM imidazole. For the elution of bound protein, the imidazole concentration was raised to 200 mM, and protease inhibitors were omitted. CCL16-containing fractions were pooled, incubated with PreScission protease (GE Healthcare Life Sciences, Solingen, Germany) for 3 h at room temperature, and dialysed overnight with 50 mM sodium dihydrogen phosphate, 500 mM sodium chloride, and 10% (*v/v*) glycerol, pH 8.0 (via sodium hydroxide). Final purification was achieved by size exclusion chromatography on a HiLoad 26/60 Superdex 75 pg column (GE Healthcare Life Sciences, Solingen, Germany) equilibrated with the same buffer; CCL16 eluted with a peak retention volume of 186 mL. The CCL16 identity and purity were verified by CE-MS analysis. For use in crystallisation experiments, the protein solution was subsequently concentrated by ultrafiltration.

### 2.10. Crystallisation and Diffraction Data Collection

Screening for crystallisation conditions was carried out at 293 K, using a robotic system (Freedom EVO; Tecan, Männedorf, Switzerland) in a sitting-drop setup with an initial protein concentration of 2.2 mg/mL. A single crystal formed in the presence of 1.6 M sodium citrate (pH 6.5) as a precipitating agent. For cryo-cooling, the crystal was directly mounted in its mother liquor. Diffraction datasets with a maximum resolution of 1.45 Å were recorded at 100 K and an X-ray wavelength of 0.979 Å, using the beamline ID23-1 of the European Synchrotron Radiation Facility (ESRF, Grenoble, France) equipped with a PILATUS 6M detector (Dectris, Baden-Daettwil, Switzerland). Data were processed using XDS and XSCALE [22].

### 2.11. Structure Determination

The CCL16 crystal belonged to space group C2, with two protein chains in the asymmetric unit. Initial phases were determined by molecular replacement using MOLREP [23] with a single native dataset and the chemokine domain of human CCL14 (PDB-ID 2Q8T [24]) as a search model. Missing parts of the structure were built interactively using Coot [25], and the model was iteratively improved by reciprocal space refinement in phenix.refine [26] and rebuilding in Coot. For the statistics of data collection and refinement, refer to Table 1. The final model contains residues 8–74 of chain A and 8–75 of chain B, corresponding to a Matthews coefficient of 2.0 Å^3^/Da and a solvent content of 39%. Validation with MolProbity [27] and Coot revealed good geometry with all of the residues in the allowed regions of the Ramachandran plot and no rotamer outliers.

### 2.12. Mass Spectrometry (MS)

The CCL16 samples were subjected to capillary electrophoresis (CE) using an Agilent 7100 capillary electrophoresis system (Agilent Technologies, Waldbronn, Germany). The capillaries (inner diameter 50 µm, length 61.3 cm) were coated with the neutral capillary coating N-acryloylamido ethoxyethanol, as described previously [28,29]. An aqueous running buffer containing 0.3 M formic acid was used. Samples were injected for 5 s at 100 mbar and separations were carried out at +30 kV (equals 489 V/cm). Before each run, the capillary was flushed with a running buffer for 300 s. An Agilent 6550 Accurate-Mass Q-ToF-MS (Agilent Technologies, Waldbronn, Germany) was used for MS detection. The CE was coupled to the mass spectrometer via a coaxial sheath liquid interface. An Agilent isocratic pump 1260 (Agilent Technologies, Waldbronn, Germany) delivered the sheath liquid, a 1:1 mixture of water and 2-propanol containing 0.01% (*v/v*) formic acid, at a flow rate of 4 µL/min. The nebuliser pressure was set to 0.21 bar and the drying gas flow to 11 L/min at 150 °C. A fragmentor voltage of 175 V, a capillary voltage of 4000 V, and an octopole voltage of 750 V were used. iFunnel parameters were as follows: the exit differential current was set to 50 V, and the reference high pass and low pass were set to 200 V and 100 V, respectively. The mass range was set to m/z = 100–3000 and the data acquisition rate was 2 spectra/s. For MS calibration, we used the G1969-85000 Q-ToF standard calibration tune mix from Agilent Technologies (Waldbronn, Germany). This standard tune mix contains betaine and phosphazine derivatives for mass calibration.

### 2.13. Molecular Dynamics (MD) Simulations

In order to obtain a starting model for MD simulations, terminal residues and side chain atoms absent from the crystal structure were built with MODELLER [30] v. 9.14. A set of twenty models was generated for each chain using the DOPE-HR algorithm, and the four dimer models with the lowest energies were selected for multiple simulated annealing (SA)-MD runs aiming at the further optimisation of terminal configurations [31]. All SA-MD runs were performed using GROMACS [32] v. 5.0.4, with the Amber ff99SB*-ILDN [33] force field and the TIP3P [34] explicit water model. The protonation states of all titratable residues were assigned to a pH of 7.4 based on the H++ analysis [35]. Disulphide bridges were defined for the C14-C37 and C15-C53 pairs of residues. The protein was centred in a dodecahedral box, at least 10 Å away from each of the box edges, and solvated with around 16,500 water molecules. The net charge of the system was neutralised with chloride anions. The cut-off for the short-range non-bonded interactions was set to 9 Å, while the long-range electrostatic interactions were treated with the particle mesh Ewald method [36] in combination with periodic boundary conditions. Energy minimisation of the system was performed in two steps: (1) a steepest descent minimisation with a convergence criterion of 500 kJ mol^−1^ nm^−1^, followed by (2) a conjugate gradient minimisation with a convergence criterion of 100 kJ mol^−1^ nm^−1^. From each of the four minimised structures, 25 independent SA-MD runs were initiated with different starting velocities, and an integration step of 2 fs was used. The simulated annealing protocol consisted of: (1) slow heating of the system to 400 K over 1 ns, (2) equilibration at 400 K for 2 ns, and (3) slow cooling of the system to 0 K over another 2 ns, yielding 5 ns of sampling per SA-MD run. To sample only terminal motion during the SA-MD runs, all atoms belonging to residues N9–L73 were fixed using a 1000 kJ mol^−1^ nm^−2^ position restraint force constant. The structure corresponding to the final frame of each SA-MD run was energy minimised using the two-step minimisation process described above.

The stereochemical quality of the resulting 100 structures was estimated using PROCHECK [37] v. 3.5.4. Only those protein conformations where (1) all residues were within the allowed regions of the Ramachandran plot and (2) no less than 80% of the residues were in the most favoured regions were chosen for further evaluation. The folding free energy was calculated for each of the selected structures using FoldX [38] v. 4, and the three most stable structures were used in further simulations. Although having the same core structure, these protein models had very different terminal conformations (backbone r.m.s. distances of 6–11 Å). We performed a 500-ns unrestrained MD simulation at 298 K for each of the three structures (r.m.s. deviation and r.m.s. fluctuation plots are shown as Supplementary Figures S5 and S6, respectively), and as all simulations converged to a similar geometry, we decided to proceed with only one of the models. In this model, the C-terminal residues of chain A were interacting with the protein core, while chain B had its C-terminus extended away from the protein.

To explore the conformational landscape of the C-termini in more detail, we performed a Hamiltonian replica exchange (HREX)-MD simulation of this model, using GROMACS v. 4.6.7 combined with the PLUMED plugin [39], as implemented by Bussi [40]. The same preparation protocol as for the SA-MD runs was used for the HREX-MD run. After the two-step energy minimisation, the system was equilibrated in the NVT (constant particle number, volume, and temperature) ensemble for 0.2 ns. The temperature was kept at 298 K using the velocity rescale thermostat [41]. All backbone atoms were restrained with a 1000 kJ mol^−1^ nm^−2^ force constant. The equilibration was continued for another 0.8 ns in the NPT (constant particle number, pressure, and temperature) ensemble, and the Berendsen barostat [42] was used to keep the pressure at 1 bar, while the restraint force constant was slowly reduced to 10 kJ mol^−1^ nm^−2^. The final state of this equilibration procedure was the starting structure for the HREX-MD simulation.

Each replica was simulated in the NPT ensemble at 298 K and 1 bar using the velocity rescale thermostat and the Parrinello–Rahman barostat [43]. An integration time of 4 fs was used in combination with the virtual sites for all hydrogen atoms [44]. Protein bonds were constrained with the P-LINCS algorithm [45]. We simulated 12 replicas, for which the Hamiltonian scaling factors of the C-terminal residues (P74–Q97) were exponentially distributed between 1.00 and 0.60 (the exact scaling factors were 1.00, 0.96, 0.91, 0.87, 0.83, 0.79, 0.76, 0.72, 0.69, 0.66, 0.63, and 0.60). These scaling factors corresponded to temperatures between 298 and 496 K. An exchange between neighbouring replicas was attempted every 4 ps. The average acceptance ratio for exchanges was 0.30. The total simulation time per replica was 120 ns, amounting to an accumulated HREX-MD simulation time of 1.44 μs. Coordinates and energies were saved every 8 ps for each replica.

For conformational analysis, we considered only structures sampled from the HREX-MD trajectory with an unperturbed Hamiltonian (i.e., a scaling factor of 1.0). The covariance matrix was built for all C-terminal (residues 75–97) backbone atoms after fitting to the protein core (residues 8–74) backbone. The first two eigenvectors obtained from the matrix diagonalisation described more than 60% of the C-terminal motion and are named main motion 1 and main motion 2 herein. GROMACS tools, VMD [46] v. 1.9.2, and MATLAB v. R2015b (MathWorks, Natick, MA, USA) were used for data analysis.

### 2.14. Figure Preparation

Molecular representations in Figures 4 and 5 were generated with POVScript+ [47] and Raster3D [48], using secondary structure assignments determined by DSSP [49] and solvent-excluded molecular surfaces calculated with MSMS [50]. In Figure 6, depictions of conformers 1–4 were prepared using VMD. After the assignment of atomic radii and charges (pH 7.4) by PDB2PQR [51], electrostatics calculations were performed with APBS [52], assuming 150 mM of monovalent cations and anions in the bulk solvent and a temperature of 310 K. PROMALS3D [53] was used for the alignment of chemokine sequences, Theseus [54] and LSQMAN [55] for structural superposition.

## 3. Results and Discussion

### 3.1. Characteristics of CCL16 Expression

**Figure 1:**
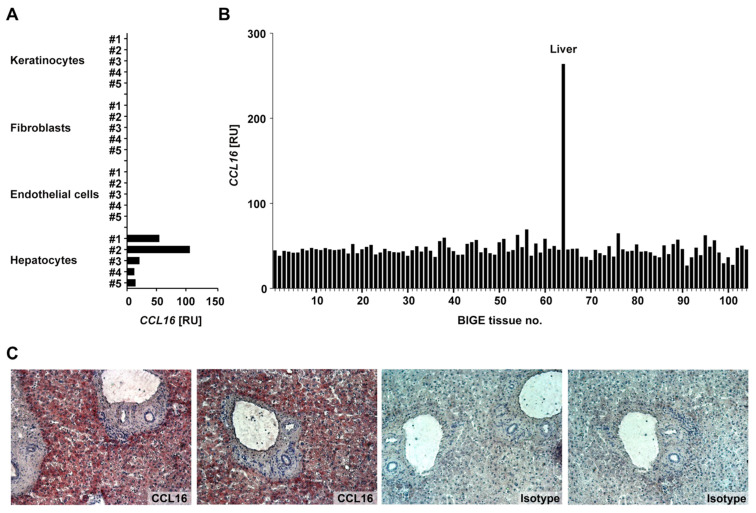
CCL16 (C-C motif ligand 16) is preferentially expressed in the liver. (**A**) The CCL16 expression of normal human primary keratinocytes, dermal fibroblasts, microvascular endothelial cells, and hepatocytes (*n* = 5 each), given as relative units (RU) normalised to 18S. (**B**) The CCL16 expression profile in the BIGE (Body Index of human Gene Expression) database representing 104 different cell types and tissues. (**C**) The immunohistochemistry of CCL16 protein expression in frozen liver sections (original magnification: ×100).

We first examined the expression of CCL16 in various human cells and tissues (Figure 1). Total RNA was extracted from primary human keratinocytes, fibroblasts, endothelial cells, and hepatocytes (*n* = 5 each) and analysed for CCL16 mRNA by a real-time PCR assay. While there was no detectable CCL16 mRNA in the structural cells of the skin, CCL16 was expressed by hepatocytes (Figure 1A). Moreover, an analysis using the Body Index of human Gene Expression (BIGE) database comprised of 104 different tissues [17,56] revealed the liver-specific expression of CCL16 (Figure 1B). On the protein level, the presence of CCL16 in the liver was confirmed by the immunohistological staining of tissue sections (Figure 1C). Together, these observations qualify CCL16 as a liver-specific chemokine, supporting the findings of Shoudai et al. [7].

**Figure 2:**
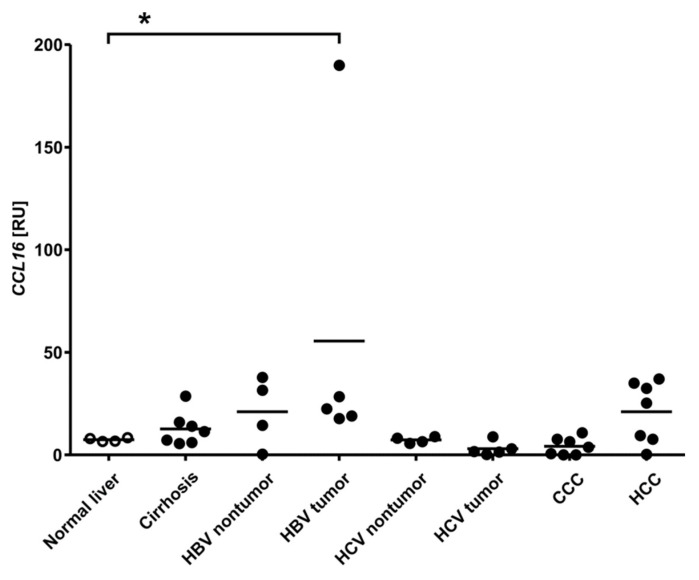
CCL16 expression in the liver occurs homeostatically. CCL16 mRNA was quantified in normal human liver tissue (*n* = 4) in comparison to livers with cirrhosis (*n* = 7), hepatitis B virus (HBV) and hepatitis C virus (HCV) infection (*n* = 4–5), as well as cholangiocarcinoma (CCC; *n* = 7) and hepatocellular carcinoma (HCC; *n* = 7). Values are expressed as relative units (RU) normalised to 18S. Measurements of individual samples and the mean of each group are shown. A Mann–Whitney U test was performed to assess statistical significance (* *p* < 0.05).

In order to explore the regulation of CCL16 expression, the respective mRNA was quantified in normal (*n* = 4) versus diseased human liver tissue (Figure 2). Intriguingly, damage of the liver, e.g., by cirrhosis (*n* = 7), HBV or HCV infection (*n* = 4–5), as well as CCC (*n* = 7) or HCC (*n* = 7), has no effect on CCL16 expression. Note that, in the group of hepatitis B-induced tumours, four out of five samples are within the range observed for other groups; the significance of the single outlier featuring extremely upregulated CCL16 is uncertain. To further corroborate our findings, we examined the response of primary human hepatocytes to inflammatory cytokines IL-1β, TNF-α, IL-6, and OSM, but did not detect significant effects on CCL16 mRNA (Supplementary Figure S1). We conclude that CCL16 expression in the liver occurs homeostatically, i.e., it is largely unresponsive to the common disease conditions examined in this study.

**Figure 3:**
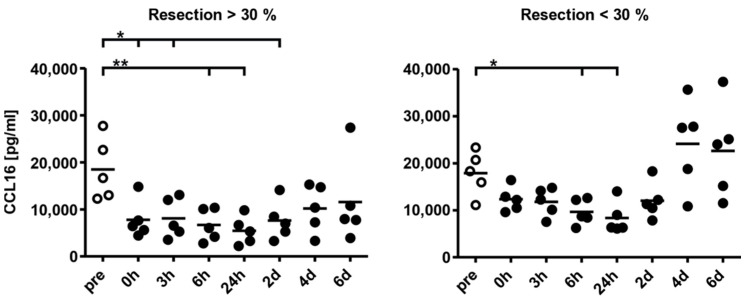
The liver is the primary source of CCL16 in human serum. CCL16 protein abundance was measured in the sera of patients who underwent liver resection. One group represents a removal of more than 30% of the liver mass, the other group had less than 30% removed. Sera were taken at the end of surgery (0 h) and at various time points thereafter and analysed by ELISA. Data are represented by individual values as well as means for each time point. A Mann–Whitney U test was performed to analyse statistical differences (* *p* < 0.05; ** *p* < 0.01).

Since the liver appears to be the primary source of CCL16 in humans, it is reasonable to assume that the global availability of chemokine may be compromised by a loss of liver tissue. To evaluate this hypothesis, CCL16 protein expression was measured by ELISA in sera from patients before partial liver resection, at the end of surgery (0 h), and at various time points thereafter (Figure 3). Patients were divided into two cohorts (*n* = 5 each): one group with resection of more than 30% of the liver (left panel), and the other group with removal of less than 30% of the liver mass (right panel). Consistent with previous reports [12,57], CCL16 is initially present in serum at relatively high concentrations (approx. 20 ng/mL). In the first group, CCL16 concentration is already significantly reduced after surgery and the mean value continues to decrease, reaching its lowest level after 6–24 h. A significantly reduced abundance of CCL16 can still be detected after two days, and serum concentration does not appear to fully normalise until day six. In the second group, CCL16 tends to be reduced as well, gaining significance at 6–24 h after surgery. Normal serum levels are reached after four days. As expected, the decrease in CCL16 serum levels thus depends on the amount of tissue removed. In addition, our data suggest a certain upregulation of CCL16 expression after surgery, since the increase observed starting from day two in both groups cannot be explained by liver regeneration. This upregulation presumably occurs in hepatocytes, but contributions from other sources cannot be excluded.

### 3.2. Structure of CCL16

In view of the unusual biological properties of CCL16, we set out to investigate the protein using biophysical methods, with a focus on its three-dimensional structure. To this end, human CCL16 fused to an N-terminal hexahistidine tag was expressed in *E. coli* SHuffle T7 cells. This strain was selected since it lacks two cytoplasmic reductases, thioredoxin reductase and glutathione oxidoreductase, allowing for the formation of disulphide bonds in cytoplasmic proteins due to a diminished reduction potential. In addition, the expression of a disulphide isomerase is expected to facilitate the establishment of the correct bonding pattern in proteins with multiple disulphide bridges. CCL16 purified from bacterial lysates turned out to be poorly soluble at a low to moderate ionic strength, with a strong propensity to form aggregates. In the presence of 500 mM sodium chloride, however, size exclusion chromatography yielded a single peak with an apparent molecular mass of about 20 kDa (determined after calibration with a set of standard proteins in the same buffer). This behaviour of CCL16 is reminiscent of its paralog CCL5, which also requires elevated ionic strength to prevent precipitation [58]. Given a theoretical mass of 11.4 kDa for a single chain, these observations suggest that (1) in solution, CCL16 exists as a dimer exhibiting a tendency to assemble into higher-order oligomers, and (2) polar or ionic interactions may play a significant role in the formation of these oligomers.

The molecular mass of purified CCL16 was measured by MS coupled to CE. Despite the high ionic strength of the sample, a direct analysis of the protein solution containing 500 mM sodium chloride was possible (Supplementary Figure S2). Sodium was baseline-separated from CCL16, allowing for the accurate determination of the molecular mass without ion suppression. While the amino acid sequence of full-length CCL16 in a reduced state corresponds to a theoretical monoisotopic mass of 11,436.06 Da and an average mass of 11,443.35 Da, we observed masses of 11,432.02 Da and 11,439.9 Da, respectively (Supplementary Figures S3 and S4). This confirmed that CCL16 was present with two intramolecular disulphide bonds, each amounting to a mass difference of about −2 Da.

Crystallisation trials with human CCL16 were carried out using a broad repertoire of screening solutions; a positive result was observed for a single condition containing sodium citrate as a precipitating agent. The crystal displayed isotropic diffraction extending to a resolution of 1.45 Å. Structure determination using the molecular replacement method was straightforward, revealing the presence of two chains per asymmetric unit. Interpretable electron density was discernible for residues 8–74 and 8–75 of chains A and B, respectively.

**Figure 4:**
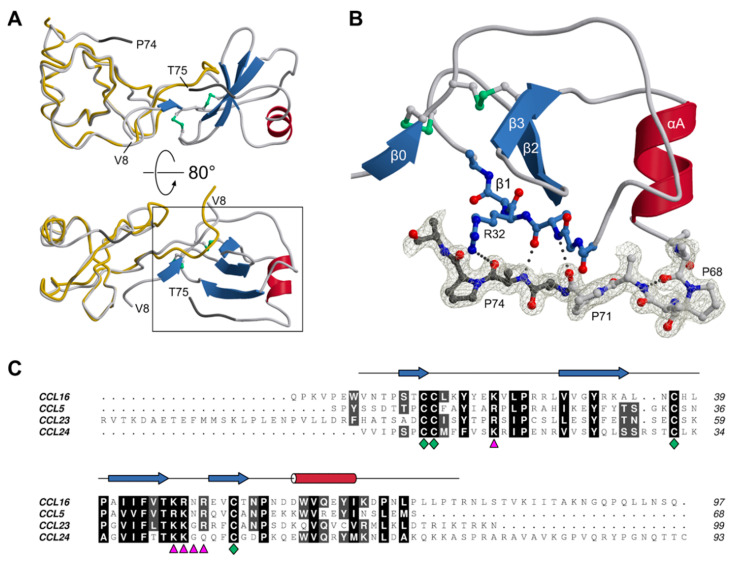
The crystal structure of human CCL16. (**A**) CCL16 forms a canonical CC-type dimer. One of the protomers (chain B, right) is shown in ribbon representation, the other (chain A, left) as a Cα trace, on which the prototypical family member CCL5 (PDB-ID 1U4L [60], chain A, yellow) is superimposed for comparison. The C-terminal extension relative to CCL5 is coloured dark grey. (**B**) Close-up view of chain B, corresponding to the boxed area in (**A**), with a focus on the C-terminal extension and its interaction with the chemokine core domain. In the segments shown in all-atom mode, most side chains have been truncated for visual clarity. Electron density (2*mF_o_*-*DF_c_* synthesis, contoured at 1.2 σ) is displayed for residues 67–75; hydrogen bonds stabilising the conformation of the terminus are indicated by dotted lines. (**C**) Sequence alignment of CCL16 with other human CC-type chemokines, including CCL23 and CCL24, which carry unique extensions at their N- and C-termini, respectively. The degree of sequence conservation is indicated by shading; green diamonds mark the invariant cysteine residues, and purple triangles identify the BBxB motif in the β2-β3 loop and an additional basic residue in the N-loop, both of which are implicated in GAG binding. The secondary structure of CCL16 (chain B of our crystal structure) is depicted above the alignment.

The two CCL16 chains constituting the asymmetric unit of the crystal were found to be very similar, with r.m.s. distances of 0.64 Å for the main-chain atoms and 1.07 Å for all non-hydrogen atoms. Regarding its three-dimensional structure as well as its dimeric arrangement, human CCL16 resembles CC-type chemokines characterised previously [59]. The protein belongs to the α+β class, with a core domain comprising a three-stranded antiparallel β-sheet and a single α-helix (Figure 4A). In addition, each chain features an extended N-terminus, which is involved in dimerization via a small intermolecular β-sheet and is connected to strand β1 by the so-called N-loop. It is noteworthy that the electron density clearly reveals cystine entities at the expected positions, confirming the utility of the engineered *E. coli* strain for the expression of disulphide-bonded proteins. A distinctive feature of CCL16 with respect to related proteins concerns its C-terminal segment. Instead of the final helical turn present, e.g., in CCL5, CCL16 contains a type-I β-turn, causing the subsequent stretch to extend perpendicularly from the helix (Figure 4B); in fact, it orients in parallel to strand β1, to which it is attached by three hydrogen bonds, and thus appears to roughly point towards the dimer interface. This conformation is consistently observed in both copies present in the asymmetric unit: after superposition of the core domains (residues 8–66), the terminal stretches (residues 67–74) align with a r.m.s. distance of 0.72 Å.

**Figure 5:**
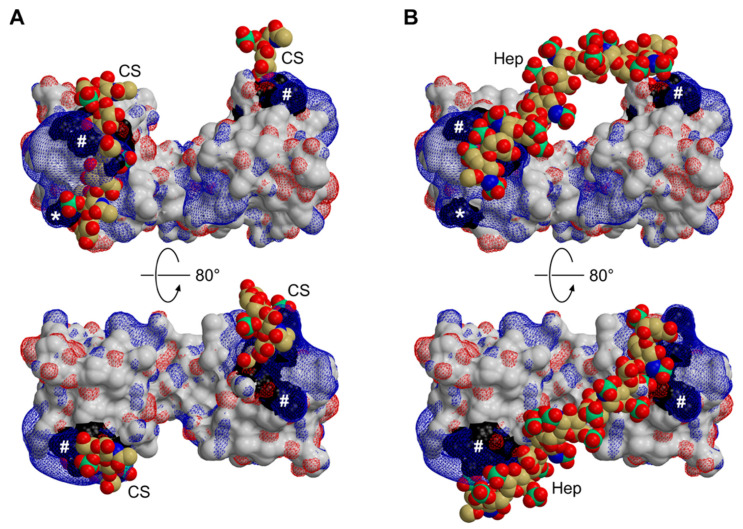
Potential GAG interaction modes of CCL16. The crystal structure of CCL16 (surface representation, oriented as in Figure 4A) is shown in the context of two published models featuring short mucopolysaccharide chains docked to CCL5. The chondroitin sulfate (CS, panel **A**) and heparin (Hep, panel **B**) moieties are drawn in space-filling mode; black patches on the CCL16 surface indicate basic residues K47, R48, and R50 of the BBxB motif (#) as well as K21 (*), suggested to mediate this interaction by analogy to CCL5. The electrostatic potential of CCL16 is contoured at +3 kT/e (blue) and −3 kT/e (red).

The remainder of the C-terminal extension (23 and 22 residues in chains A and B, respectively) could not be traced in the electron density (Figure 4C). Indeed, the sequence-based analysis predicts this segment to be disordered. Possible implications of terminal dynamics with respect to the physiological function of CCL16 are discussed below. Akin to related chemokines, CCL16 is a highly basic protein, with an isoelectric point of 9.4 and a net charge of +7.0 at pH 7, as estimated from its primary structure (http://protcalc.sourceforge.net). Clusters of positively charged side chains are thought to determine the affinity of chemokines for different types of glycosaminoglycan. Indeed, CCL16 contains a BBxB motif [61], with B denoting a basic residue, in the 40s loop (^47^KRNR^50^), which has been confirmed to be a principal GAG binding site in several CC-type chemokines [62,63,64]. In the case of CCL5, a secondary epitope centred on R17 in the N-loop (corresponding to K21 in CCL16) has been identified, and two different models of CCL5-GAG complexes have been recently published [65,66]. Using NMR spectroscopy, Deshauer et al. have mapped contacts with hexasaccharide fragments of chondroitin sulphate [65]; their results indicate that GAG molecules interact with the 40s loop and the N-loop of one CCL5 chain, as well as the nearby N-terminus of the second chain. In contrast, Singh et al. applied automated docking followed by MD simulation to investigate the binding of a heparin dodecasaccharide [66]; the final model features a single GAG chain simultaneously contacting the 40s loops of both chemokine protomers. As illustrated in Figure 5, the structure and electrostatic properties of the CCL16 core are compatible with either mode of GAG binding. Combining the two models, a linear sulphated polysaccharide might, in fact, cover the ^47^KRNR^50^ motif as well as the K21 region on both subunits of the dimer. It should be kept in mind, however, that the chemokine-GAG interaction may be altered to a varying extent by the formation of higher-order structures. In the case of CCL5, for instance, two different modes of oligomerisation have been proposed based on experimental evidence; in one of these, [67], the canonical BBxB motif is solvent-exposed and thus accessible for binding GAG chains, while in the other, [58], this site is mostly buried and an alternative basic stretch in the 50s loop may take over its function. As noted above, CCL16 displays a propensity to aggregate at moderate ionic strength and in the absence of GAGs, akin to CCL5; whether the two molecules exhibit similar modes of self-association, however, remains uncertain. Irrespective of the oligomeric state, chemokine-GAG interactions are thought to be intrinsically dynamic [65,68]. It thus seems unlikely that all sites will be occupied simultaneously and in a well-defined conformation; instead, they should be thought of as contributing to the overall avidity in a fluctuating network of interactions.

### 3.3. C-Terminal Dynamics

**Figure 6:**
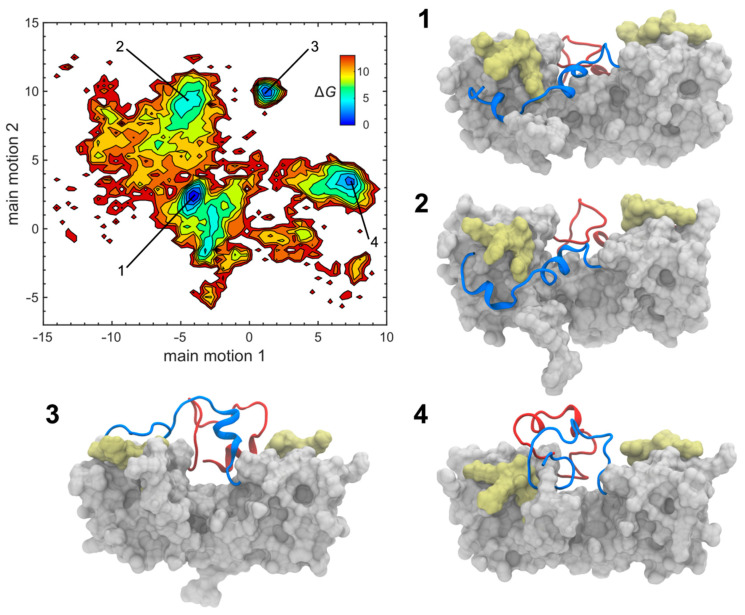
The free energy surface of CCL16. The free energy ΔG (given in kJ mol^−1^ relative to the lowest energy state) is plotted against the two main motion vectors representing the C-terminal flexibility. Structures 1–4 correspond to the free energy minima labelled in the diagram. Conformer 1 represents the global minimum, i.e., ΔG = 0 kJ mol^−1^. Conformers 1 and 2 have their C-termini positioned laterally to the protein core, interacting with the GAG binding domain of the opposite chain. In conformers 3 and 4, the C-termini interact with each other above the groove formed between the two CCL16 chains. Residues Q1–L73 are shown as a molecular surface with the GAG binding segments (K47–R50) highlighted in yellow. C-termini (P74–Q97) are rendered as ribbons, where chains A and B are coloured blue and red, respectively. For further structural details consult Supplementary Figures S7–S10.

Structural insights into the dynamic interactions of the CCL16 C-terminus were obtained from HREX-MD simulations. The large-scale collective motion of the C-terminal backbone was evaluated via principal component analysis. The first two eigenvectors turned out to account for more than 60% of the C-terminal motion. The 2D free energy landscape plotted along these two vectors (Figure 6) revealed four distinct energy minima, where conformer 1 represents the structure of the C-terminal regions at the global free energy minimum. The fact that several other minima are reachable through intermediate CCL16 conformations with higher relative free energies (up to 12.5 kJ mol^−1^) indicates that the terminal residues are highly dynamic. It should be noted that, if one were interested in the exact free energies of these intermediate conformations and the details of the transitions between them, further simulations such as umbrella sampling or metadynamics MD simulations would need to be performed. While the C-terminal residues adopt different folds in all conformers (1–4), the remainder of the protein is rather stable, with a pairwise backbone atom r.m.s. distance for residues N9–L73 on the order of 1.2 Å. The GAG binding segment (^47^KRNR^50^), however, is more flexible than the rest of the protein core.

Conformers 1 and 2 are characterised by the C-termini being oriented laterally to the protein core. In these conformations, the C-terminal residues can form stabilising interactions with the GAG binding domain of the opposite chain (e.g., K87-R50 and Q92-R48 in conformer 1). These interactions become stronger with increasing values of the main motion vector 2, which brings the C-termini even closer to the BBxB motifs (conformer 2). The nature of the interactions that stabilise this conformation involve mostly hydrogen bonds between the highly charged β2-β3 loop (^46^TKRNRE^51^) and polar C-terminal residues (K87, N88, Q90, Q92, N95, and S96). These interactions are, however, not very robust and static, which allows for most of the C-terminal residues (N77–Q97) to form short-term contacts with the GAG binding domain. Hence, the free energy surface appears rather flat, indicating structural flexibility.

The main motion vector 1 represents a significant dihedral rearrangement in the ^78^LST^80^ regions of both chains. The movement to the positive side of this vector yields conformers 3 and 4, in which both C-termini interact with each other above the central groove of the CCL16 dimer. A complex and dynamic network of hydrogen bonds is formed among the polar C-terminal residues (R76, N77, S79, T80, K82, T85, K87, N88, Q90, Q92, N95, S96, and Q97). In principle, this could allow for many different three-dimensional arrangements of the C-termini. However, the free energy surface reveals that only certain structures are allowed, corresponding to the minima for positive values of the main motion vector 1. Moreover, this type of C-terminal arrangement has a smaller overall probability than that in conformers 1 and 2. Apart from the mutual interactions, the C-termini also form contacts with the GAG binding domains of the opposite and the same chain (conformer 4), or only of the opposite chain (conformer 3). These interactions are mostly stabilised by hydrogen bonds (e.g., R76-E51, N88-N49, G89-N49, Q90-R48, and Q92-N49).

Although much shorter than the C-terminal extensions, the N-termini of CCL16 also possess some level of flexibility; yet, they are typically pointing away from the protein core. In conformers 1 and 2, N- and C-terminal residues of the same chain interact with each other (e.g., E6-K87, E6-Q90, W7-N88, and T10-Q92), which additionally stabilises this type of structure. The N-termini are also in contact with the GAG binding domains in all four conformers. These interactions are diverse, including both hydrogen bonds (e.g., P5-R50, W7-E51, and T10-E51) and ionic interactions (e.g., E6-R50).

### 3.4. Functional Implications

**Figure 7:**
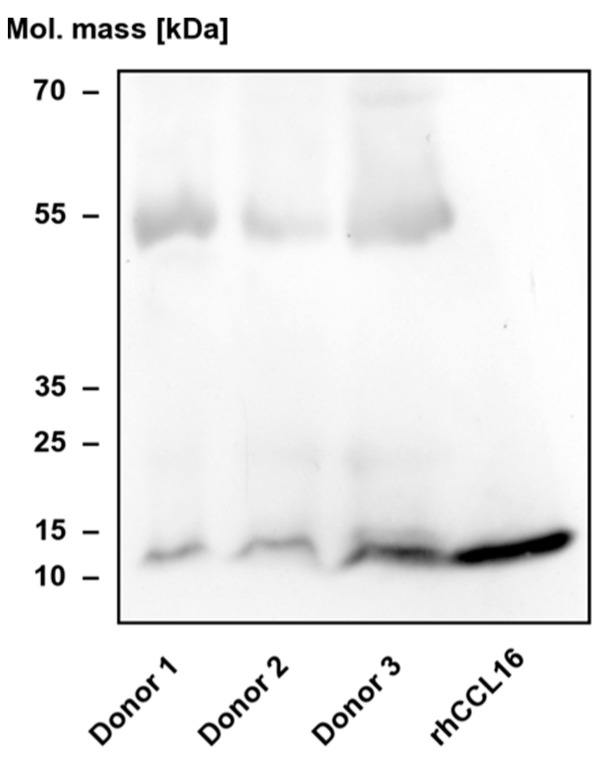
Enrichment of CCL16 from human serum. Heparin-binding proteins were enriched from the sera of human subjects (*n* = 3) via heparin affinity chromatography. Expression was determined by immunoblotting analysis using a polyclonal goat anti-human CCL16 antibody. As a control, the fourth lane contains 0.4 ng of recombinant human (rh) CCL16. The identity of the immunoreactive band with about 55 kDa apparent mass is unknown.

Taken together, the results of our HREX-MD studies indicate that the flexible termini of CCL16, in particular the C-terminal extension, show a significant propensity to reside in the vicinity of or directly interact with the BBxB motif in the β2-β3 loop, which is considered the primary binding site for acidic mucopolysaccharides. Given that the receptor binding of chemokines is usually mediated by residues of the core domain as well as the N-terminal segment, we speculate that the extended C-terminus might have evolved as an intrinsic modulator designed to interfere with GAG binding. This view is supported by the observation that truncated CCL16, which can be formed by limited proteolysis of the C-terminal segment, elutes from a heparin-sepharose matrix at a higher ionic strength than the full-length protein, indicating a higher affinity [10]. Since mucopolysaccharide binding has been found to correlate with oligomerisation in related chemokines, with both processes possibly relying on overlapping sites, the C-terminal tail of CCL16 is likely to influence self-association as well. Importantly, we found that CCL16 enriched from the sera of healthy human subjects via heparin affinity chromatography exhibits the same electrophoretic mobility as the recombinant protein used as a control (Figure 7); species with lower apparent mass were not detectable. We conclude that the circulating CCL16 pool consists primarily of the full-length cytokine.

Based on these considerations, we propose the following model of the biological life cycle of CCL16: The 97-aa protein secreted after cleavage of the signal peptide features a reduced affinity for GAGs due to the presence of its auto-inhibitory C-terminal tail. As a result, the protein circulates in the bloodstream at relatively high concentrations, but this pool is incapable of evoking strong biological responses. Local inflammatory processes, however, will usually involve the release of proteases, such as matrix metalloproteinases and cathepsins, which catalyse the terminal processing of CCL16. Due to its enhanced GAG binding, possibly coupled with an increased propensity to oligomerise, this locally activated chemokine fraction may now establish haptotactic gradients inducing, e.g., leukocyte, migration. In contrast to the canonical mechanism established for short chemokines, gradient formation would therefore be dominated by the local activation, rather than the local secretion, of the protein. Note that this model is also consistent with the liver being the primary source of CCL16. While unusual for a chemokine, it is analogous to a plethora of plasma proteins (such as haemostatic factors) which are secreted by hepatocytes as pro-forms ready to be activated rapidly as required. The CCL16 levels in human serum would thus reflect the balance between the synthetic capacity of the liver (a function of both the mass of intact tissue and its gene expression) on the one hand and peripheral consumption on the other. This is in accordance with our observation that the loss of hepatic parenchyma results in a temporary decline of CCL16 serum levels, which can be compensated by the upregulation of expression, whereas chronic liver disease, as long as sufficient functional tissue is left, does not have a significant impact. Experiments in mice indicate that the development of cirrhosis can actually be delayed by CCL16 via the inhibition of hepatic stellate cells, suggesting that this chemokine may exert a unique disease-limiting role in the liver [69]. Notably, a recent evaluation of a large number of HCC samples confirmed that mean CCL16 mRNA levels were essentially unchanged with respect to non-tumour tissue, but also revealed a low-populated tail of the distribution extending towards very high figures, which was not observed in the reference group [70]. These samples may reflect cases with more significant local inflammation, possibly leading to CCL16 induction in non-hepatocytes, or with overproduction by the tumour cells themselves; our HBV-related tumour cohort featuring a lower number of specimens with a single outlier (Figure 2) may actually be sampling the same underlying distribution. Remarkably, a recent survey of five independent proteomics studies in COVID-19 patients revealed a consistent reduction in CCL16 in blood samples [71]. While the underlying mechanisms have not been investigated, the systemic nature of the disease, potentially involving significant damage to liver tissue [72], would appear consistent both with reduced synthesis in hepatocytes and with enhanced turnover in the periphery.

## Figures and Tables

**Table 1:** Data collection and refinement statistics (r.m.s., root-mean-square). Values in parentheses refer to the highest-resolution shell.

	**Human CCL16** **(PDB-ID 5LTL)**
**Data Collection**	
Space group	C 1 2 1
Cell dimensions	
a, b, c {Å}	136.35, 24.59, 37.97
α, β, γ {°}	90.00, 93.12, 90.00
Resolution range	37.91–1.45 (1.49–1.45)
Wilson B factor {Å^2^}	20.4
CC_1/2_	99.8 (88.3)
R_meas_ {%}	6.3 (59.1)
I/σI	12.6 (2.3)
Completeness {%}	99.5 (99.6)
Multiplicity	5.0 (5.1)
**Refinement**	
Resolution range	37.91–1.45
No. reflections	22,637
R_work_/R_free_	0.171/0.198
Mean B {Å^2^} (no. atoms)	
Protein	29.3 (1140)
Ions/ligands	55.1 (7)
Water	39.1 (91)
R.m.s. deviation	
Bond lengths {Å}	0.006
Bond angles {°}	1.049
Ramachandran plot {% res.}	
Favoured	97.7
Allowed	2.3
Outliers	0

## Data Availability

Atomic coordinates and structure factor amplitudes for the crystal structure of human CCL16 have been deposited in the Protein Data Bank (www.wwpdb.org (accessed on 5 October 2022)) with accession code 5LTL (doi: 10.2210/pdb5LTL/pdb).

## Supplementary Materials

The following supporting information can be downloaded at: https://www.mdpi.com/article/10.3390/biom12111588/s1, Figure S1: Cytokine effects on CCL16 expression in hepatocytes; Figure S2: Ion electropherograms for CCL16 CE-MS analysis; Figure S3: Charge states of CCL16; Figure S4: Deconvoluted mass spectra of CCL16; Figure S5: Backbone r.m.s. deviation during CCL16 MD simulations; Figure S6: Cα r.m.s. fluctuation during CCL16 MD simulations; Figure S7: CCL16 conformer 1; Figure S8: CCL16 conformer 2; Figure S9: CCL16 conformer 3; Figure S10: CCL16 conformer 4.
