# Supplementary material for: Structure and Dynamics of Human Chemokine CCL16—Implications for Biological Activity
Source: Biomolecules. 2022 Oct 28;12(11):1588. doi: 10.3390/biom12111588 (PMC9687583; doi:10.3390/biom12111588)
Supplement: Supplementary file 1 [file biomolecules-12-01588-s001.zip › biomolecules-1982423-supplementary.pdf]

Supplementary Information

Oliver H. Weiergräber, Dušan Petrović, Andreas Kislat, Martin Pattky, Judith Fabig, Renu Batra-Safferling, Jan Schulte am Esch, Karen Hänel, Carolin Huhn, Birgit Strodel, Bernhard Homey & Dieter Willbold

Structure and dynamics of human chemokine CCL16—
Implications for biological activity

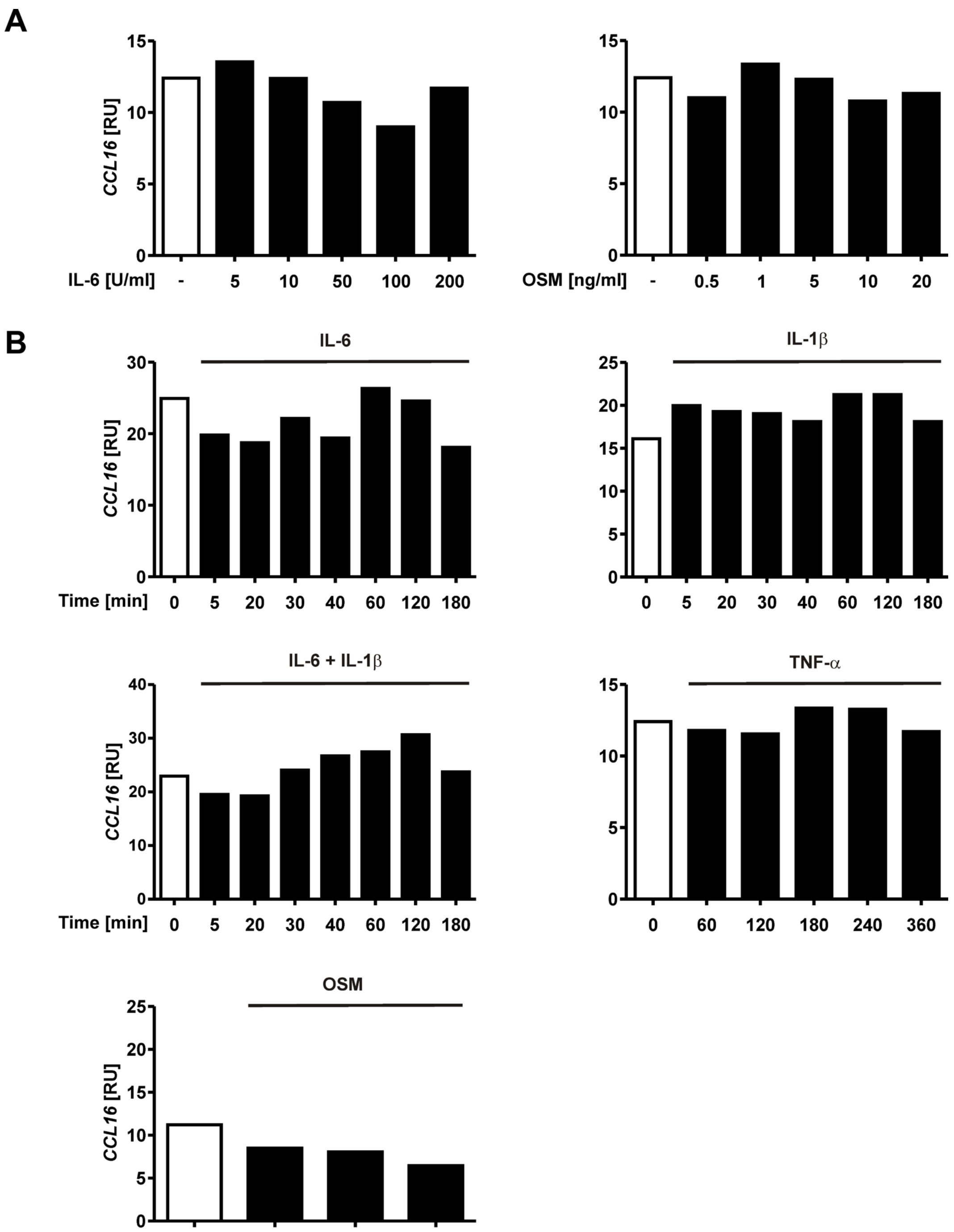

Supplementary Figure S1. Effects of different cytokines on CCL16 mRNA expression in human primary hepatocytes. (**A**) Influence of different concentrations of IL-6 and OSM on the expression of CCL16 after six hours. (**B**) Time course of CCL16 expression after addition of IL-6, IL-1β, IL-6 + IL-1β, TNF-α and OSM. Values are expressed as relative units (RU) of CCL16 in 25 ng of total cDNA normalised to 18S. Measurements of individual samples are shown, except for the last panel in (**B**), which gives means of two independent experiments.

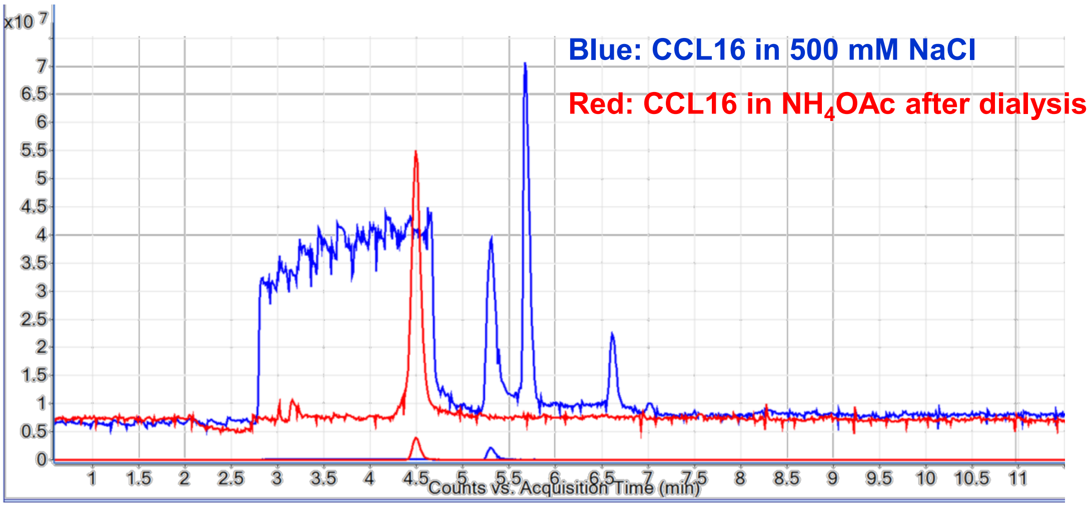

Supplementary Figure S2. Total ion electropherograms (TIC, upper traces) obtained for CCL16 CE-MS analysis and extracted ion electropherograms (EIC, lower traces) of CCL16 (m/z 1145.0144). Due to the challenging sodium chloride content in the original CCL16 solution (500 mM), a portion of the solution was dialysed to facilitate CE-MS analysis. However, analysis of the dialyzed (red) and the unmodified (blue) CCL-16 solution provide similar results, which confirms the excellent performance of the chosen CE-MS method. The good separation of CCL16 from sodium (signal at 2.75–4.75 min, compare blue TIC) and other abundant matrix components (signals at 5.7 and 6.6 min in the blue TIC) ensured good detection conditions for CCl16 without significant ion suppression.

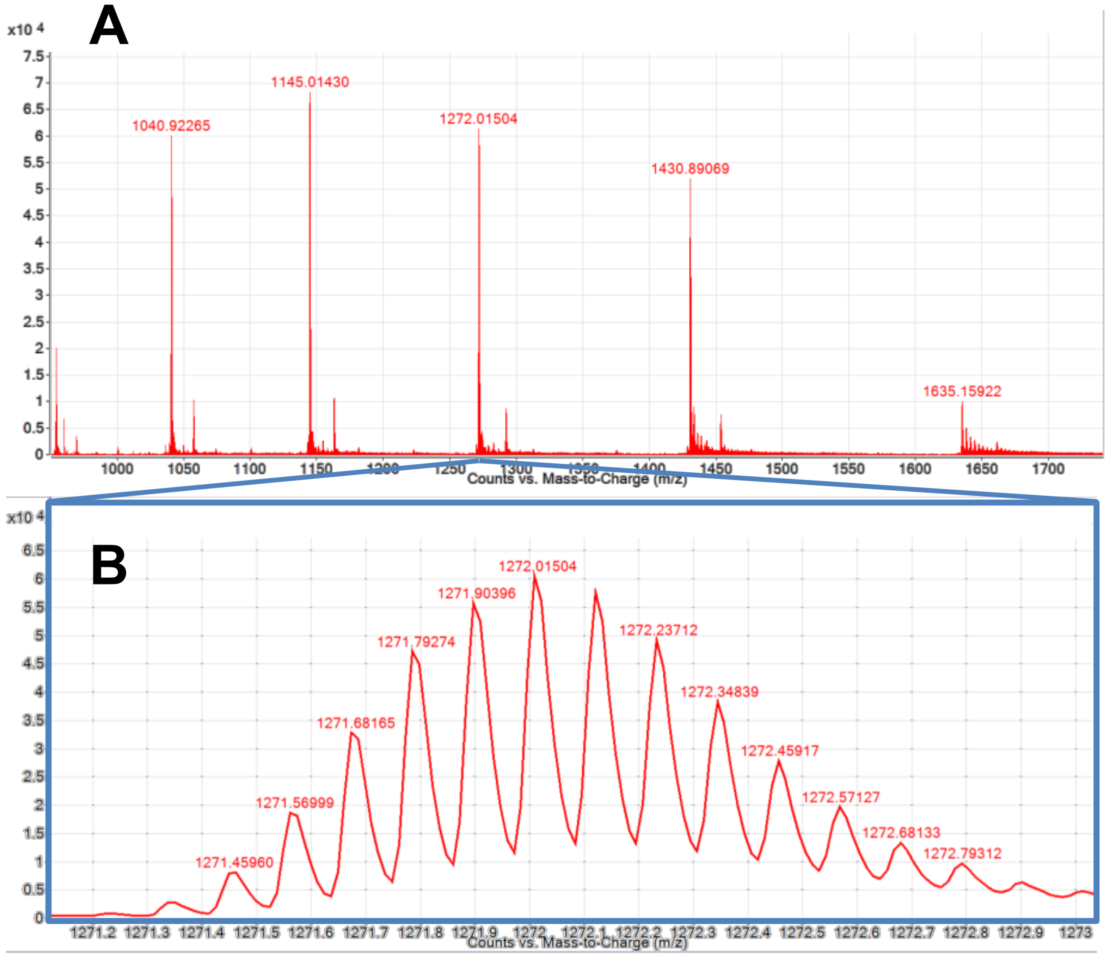

Supplementary Figure S3. Charge states of CCL16. (**A**) The six most abundant charge states of the CCL16 peptide obtained for the dialysed sample are shown (the signal at m/z 954 is not labelled). (**B**) Enlarged view on the well resolved isotope signals of the CCL16 peptide for the 9-fold charged analyte signal. Despite the high molecular mass and the high charge states, the monoisotopic mass could still be observed and used for CCL16 identification.

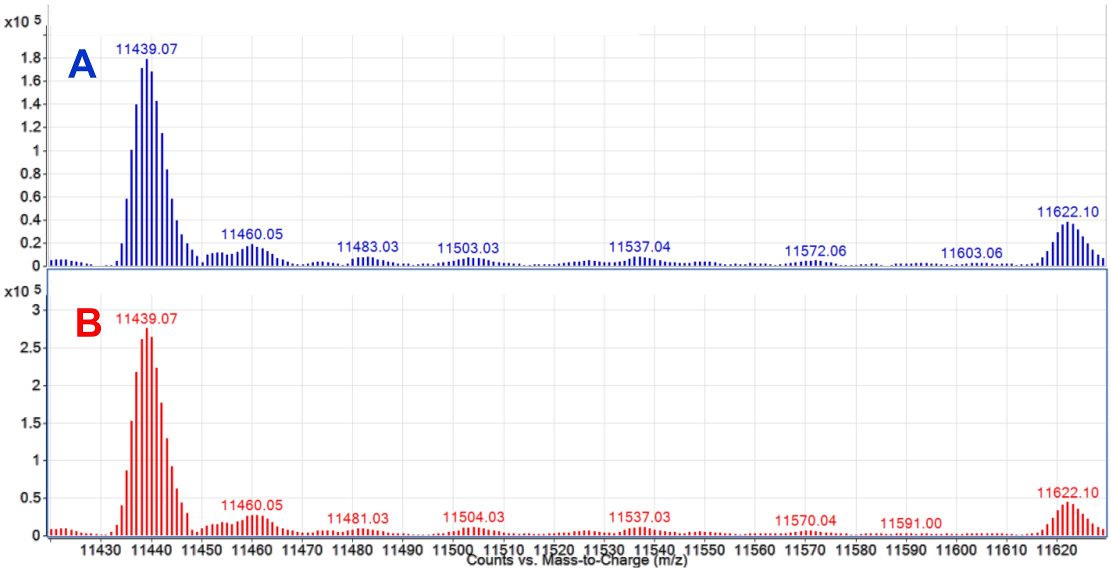

Supplementary Figure S4. Deconvoluted mass spectra of CCL16. Data are shown for the unmodified (**A**) and the dialysed (**B**) CCL16 solution. In both cases, deconvolution provides identical results, confirming CCL16 identity.

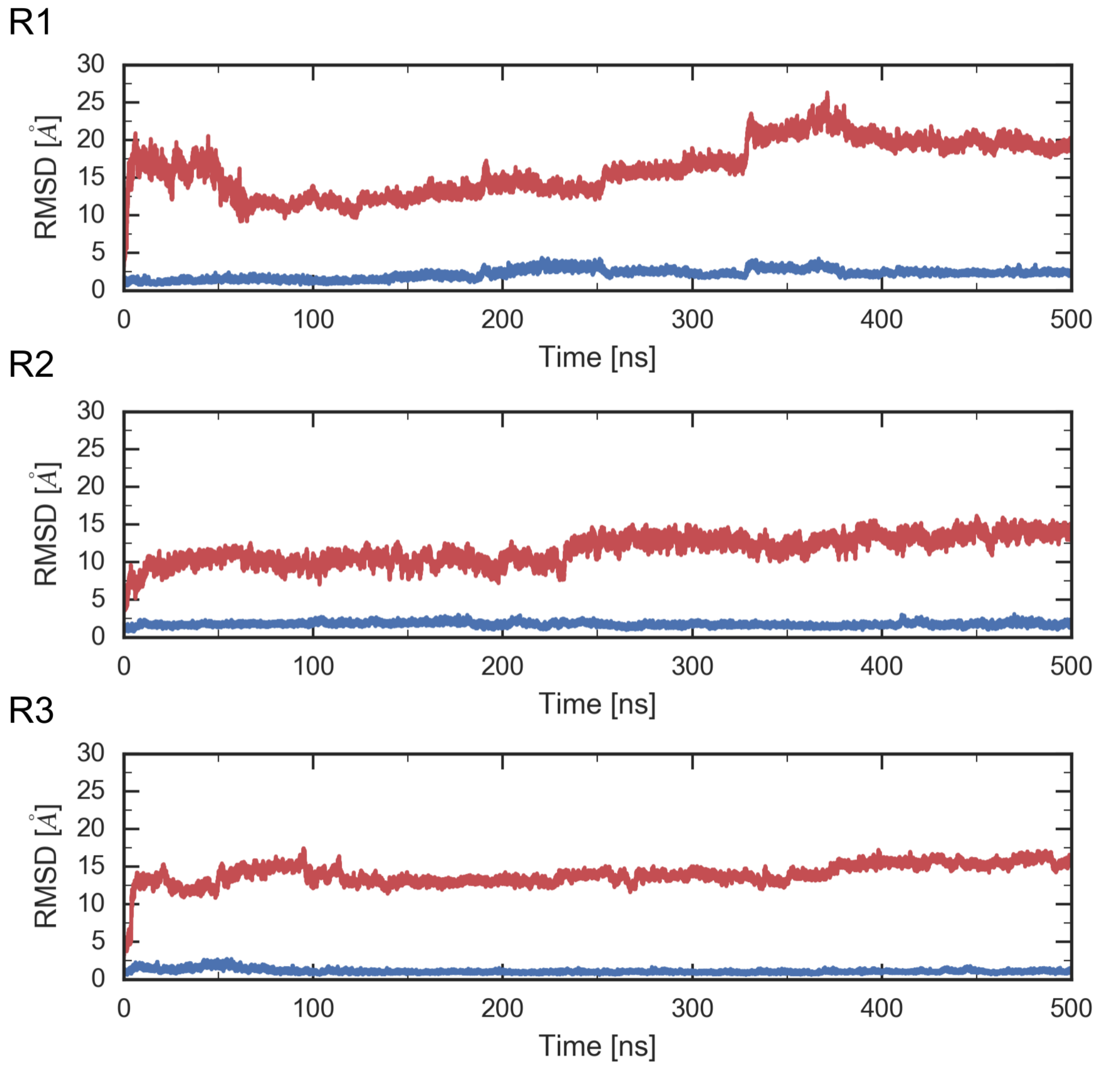

Supplementary Figure S5. Plots of backbone root-mean-square deviation (RMSD) over the three independent 500-ns MD simulations (R1, R2, and R3). The core (residues 8–74) and terminal (remaining residues) RMSDs, calculated after alignment of the core backbone atoms, are shown in blue and red, respectively. Both chains were combined for this analysis. While the core exhibits little dynamics (RMSD ≈ 2.5 Å), the termini are very flexible (RMSD > 10 Å).

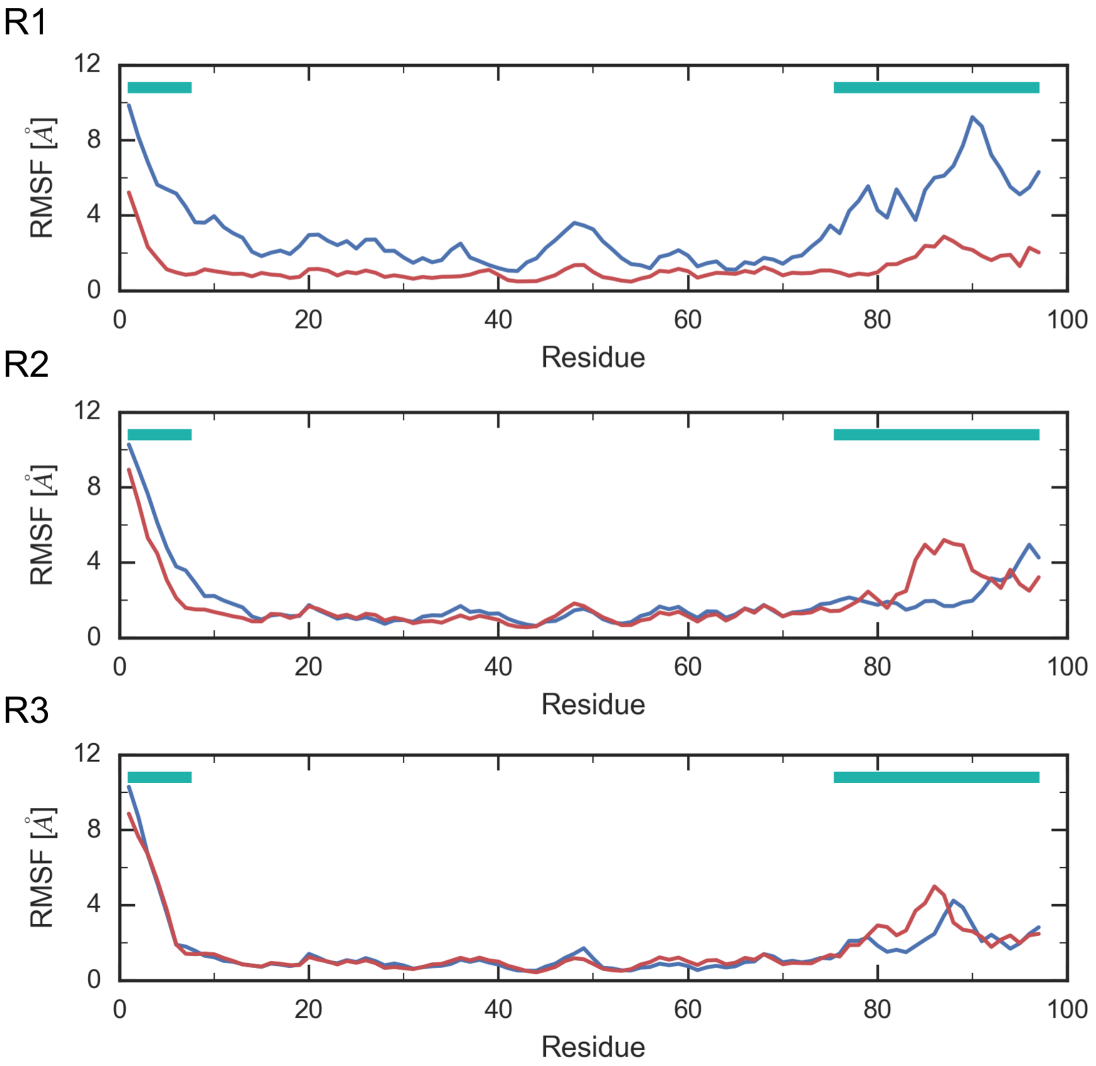

Supplementary Figure S6. Plots of Cα root-mean-square fluctuation (RMSF) over the final 400 ns of the three independent 500-ns MD simulations (R1, R2, and R3). Chains A and B are shown in blue and red, respectively. The N- and C-terminal residues, whose positions are indicated by the cyan bars on the top, are more flexible than the protein core.

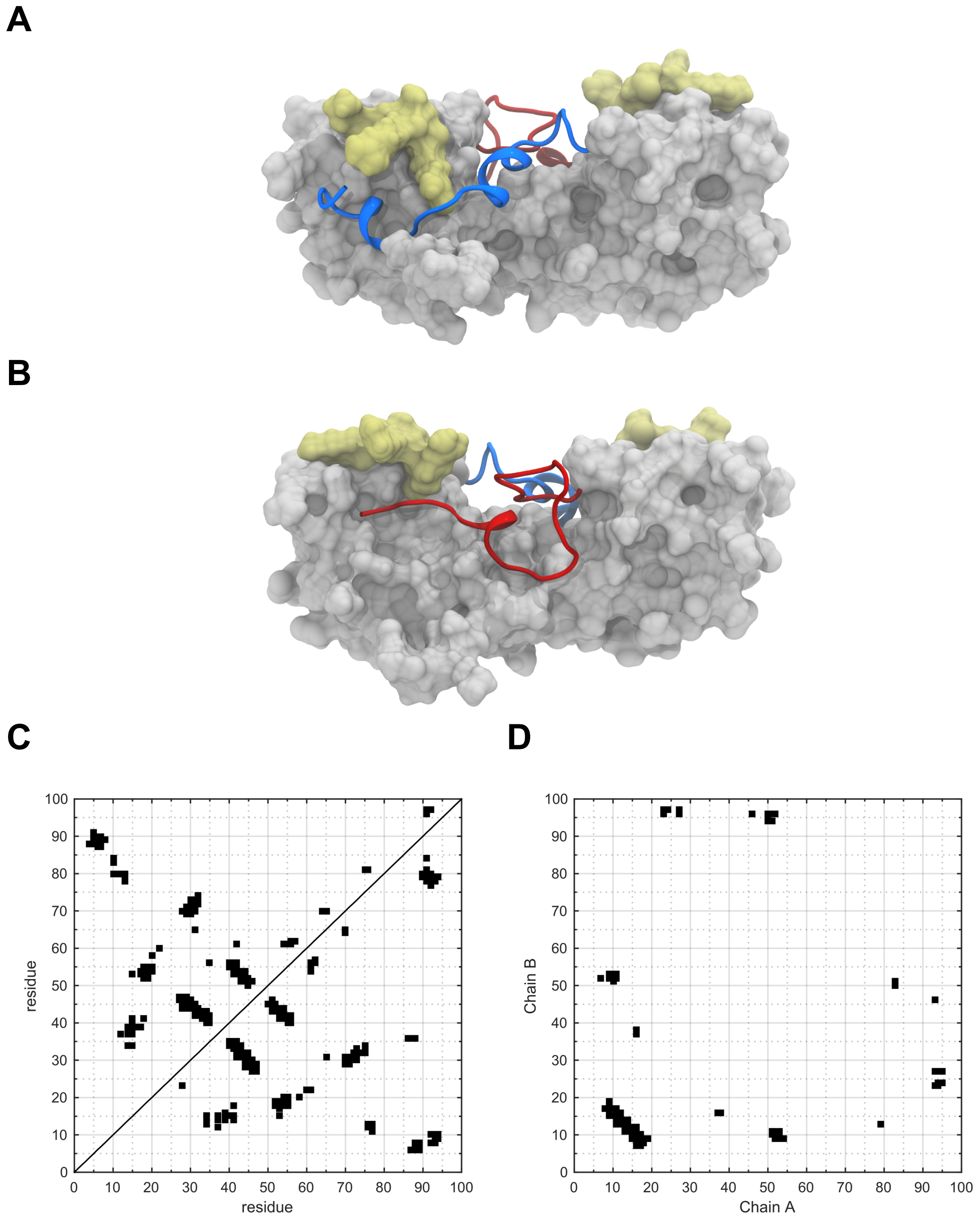

Supplementary Figure S7. CCL16 conformer 1. The dimeric structure is shown in panels (**A**) and (**B**) (180° rotation). Residues Q1–L73 are represented as a grey surface, the GAG binding regions (K47–R50) are highlighted in yellow. C-termini (P74–Q97) are rendered as cartoons, where chains A and B are coloured blue and red, respectively. Native contacts are shown in panels (**C**) and (**D**). (**C**) Contacts within a single chain (top half, chain A; bottom half, chain B). (**D**) Contacts between the chains.

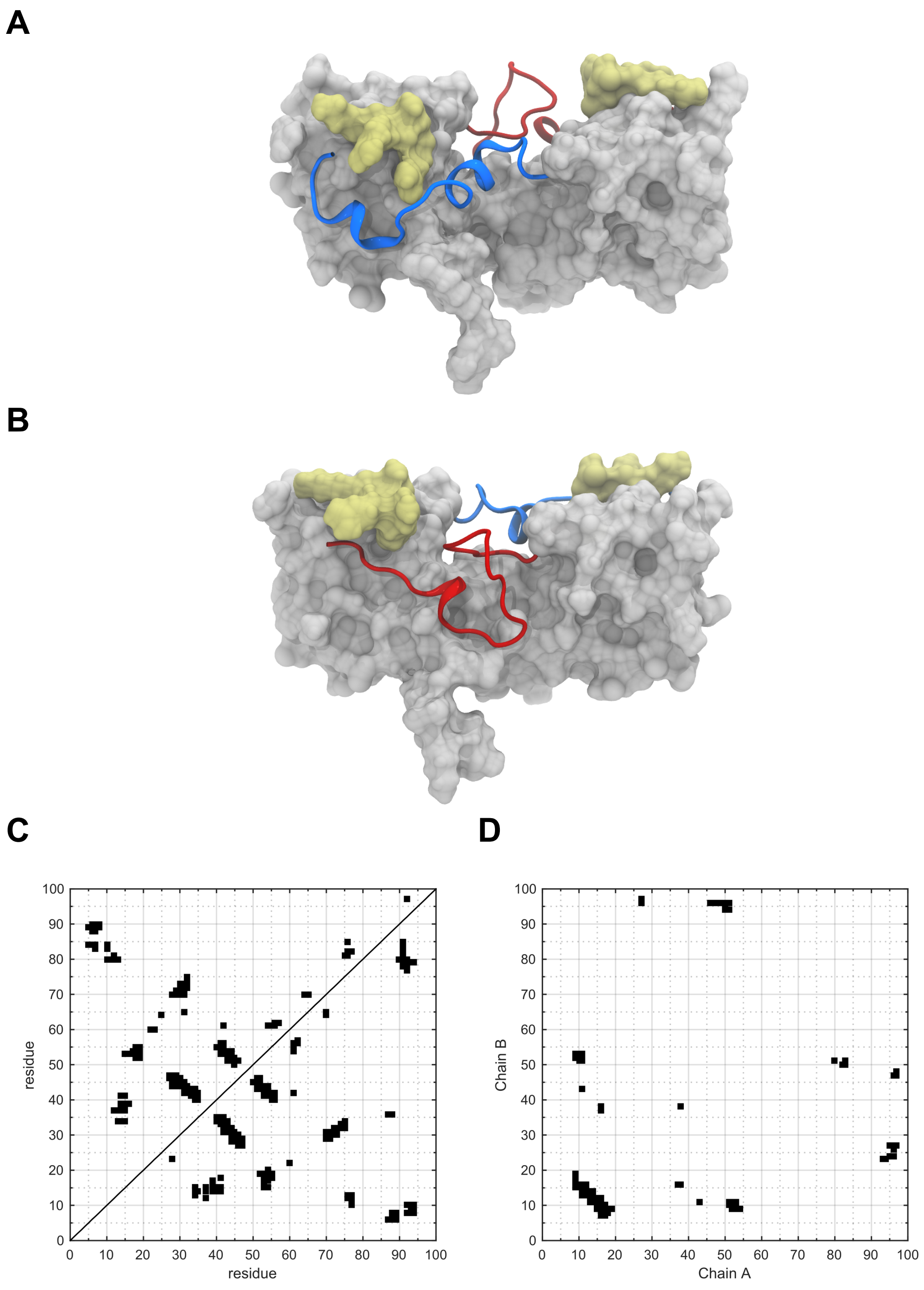

Supplementary Figure S8. CCL16 conformer 2. The dimeric structure is shown in panels (**A**) and (**B**) (180° rotation). Residues Q1–L73 are represented as a grey surface, the GAG binding regions (K47–R50) are highlighted in yellow. C-termini (P74–Q97) are rendered as cartoons, where chains A and B are coloured blue and red, respectively. Native contacts are shown in panels (**C**) and (**D**). (**C**) Contacts within a single chain (top half, chain A; bottom half, chain B). (**D**) Contacts between the chains.

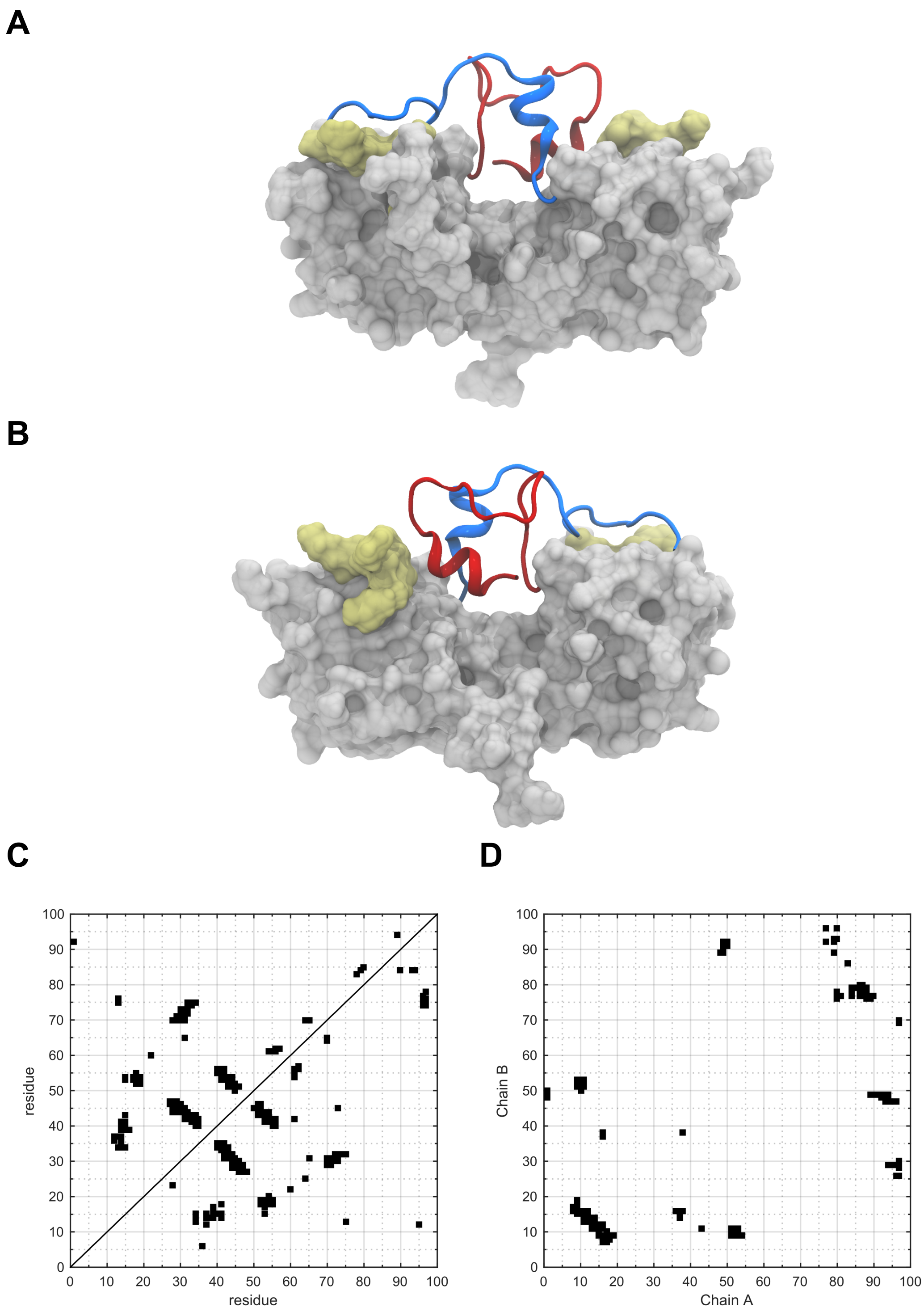

Supplementary Figure S9. CCL16 conformer 3. The dimeric structure is shown in panels (**A**) and (**B**) (180° rotation). Residues Q1–L73 are represented as a grey surface, the GAG binding regions (K47–R50) are highlighted in yellow. C-termini (P74–Q97) are rendered as cartoons, where chains A and B are coloured blue and red, respectively. Native contacts are shown in panels (**C**) and (**D**). (**C**) Contacts within a single chain (top half, chain A; bottom half, chain B). (**D**) Contacts between the chains.

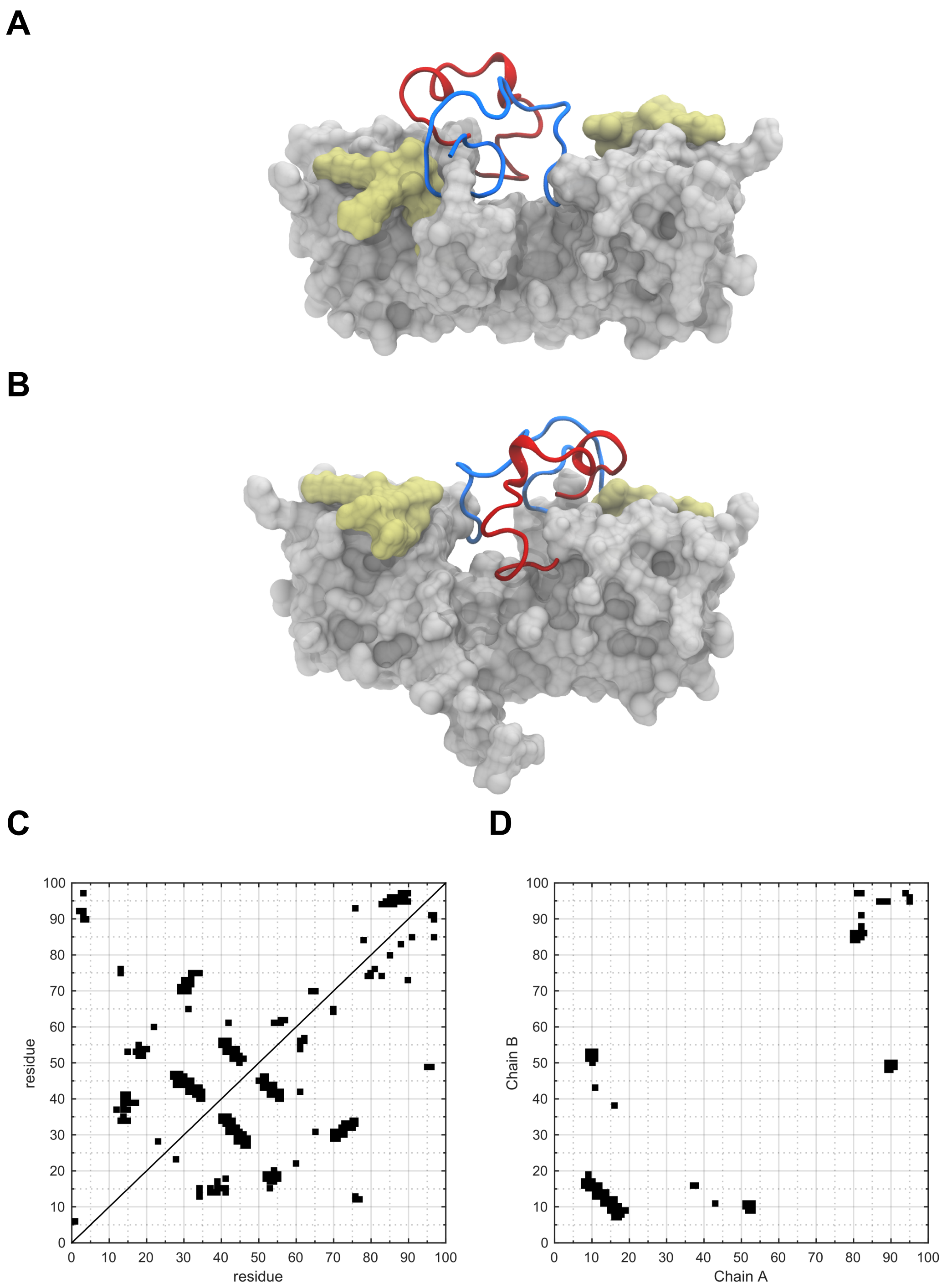

Supplementary Figure S10. CCL16 conformer 4. The dimeric structure is shown in panels (**A**) and (**B**) (180° rotation). Residues Q1–L73 are represented as a grey surface, the GAG binding regions (K47–R50) are highlighted in yellow. C-termini (P74–Q97) are rendered as cartoons, where chains A and B are coloured blue and red, respectively. Native contacts are shown in panels (**C**) and (**D**). (**C**) Contacts within a single chain (top half, chain A; bottom half, chain B). (**D**) Contacts between the chains.
